# Prognostic value of the microRNA-214 in multiple human cancers: a meta-analysis of observational studies

**DOI:** 10.18632/oncotarget.17642

**Published:** 2017-05-06

**Authors:** Yajing Feng, Fujiao Duan, Weigang Liu, Xiaoli Fu, Shuli Cui, Zhenxing Yang

**Affiliations:** ^1^ Department of Nosocomial Infection Management, The First Affiliated Hospital of Zhengzhou University, Zhengzhou, Henan, China; ^2^ Medical Research Office, Affiliated Cancer Hospital of Zhengzhou University, Zhengzhou, Henan, China; ^3^ Medical Record Statistics Office, Affiliated Hospital of Hebei University of Engineering, Handan, Hebei, China; ^4^ College of Public Health, Zhengzhou University, Zhengzhou, Henan, China; ^5^ College of Professional Study, Northeastern University, Boston, Massachusetts, USA; ^6^ Henan Key Laboratory of Tumor Epidemiology, Zhengzhou, Henan, China

**Keywords:** miR-214, cancer, prognosis, systematic evaluation

## Abstract

Previous studies showed that microRNA-214 (miR-214) may act as a prognostic biomarker of cancer. However, the available evidence is controversial. This study summarizes evidence and evaluates the prognostic role of miR-214 in various cancers. We carried out a systematic literature review and assessed the quality of included studies based on Oxford Centre for Evidence-based Medicine Criteria and Newcastle-Ottawa Scale (NOS). Pooled hazard ratios (HRs) with corresponding 95% confidence intervals (CIs) for overall survival (OS) and disease free survival/progressive free survival/recurrence free survival (DFS/PFS/RFS) were calculated to measure the effective value of miR-214 expression on prognosis. Thirteen studies were included in pooled analysis. We found that miR-214 was significantly correlated with OS (HR=2.21, 95%CI: 1.33-3.68, *P*=0.00), no significant difference was found with DFS/PFS/RFS (HR=1.73, 95%CI: 0.78-3.83, *P*=0.18) in various carcinomas. In subgroup analysis, higher expression of miR-214 was significantly associated with poor OS in Asians (HR=2.27, 95%CI: 1.09-4.73, *P*=0.00) and Caucasians (HR=2.04, 95%CI: 1.47-3.30, *P*=0.00). On the contrary, high miR-214 expression significantly predicted favorable DFS/PFS/RFS (HR=0.50, 95%CI: 0.31-0.82, *P*=0.00) in hepatocellular carcinoma (HCC) group. Our data indicates that high miR-214 could be a promising biomarker for prognosis prediction of cancer. However, further clinical studies are needed for the current insufficient relevant data.

## INTRODUCTION

MicroRNAs (miRNAs) are highly evolutionary conserved, noncoding RNAs containing about 22 nucleotides in length that participate in a variety of biological processes [1]. The 3’untranslated (3’UTR) region of target mRNAs, can be bound to complementary sequences, and lead to translational repression or down-regulation of its target mRNA translation [2], miRNAs has been shown to play a crucial important role in the process of oncogenesis and metastasis of tumor [3, 4].

The function of miRNAs is now well established in the development and progression of cancer [5], involved in regional tumor angiogenesis, cell proliferation, differentiation, migration and invasion [6, 7]. The expressions of dysregulated miRNAs profiles are associated with different types of cancer and their functions vary largely with tissue types [8]. The expression levels of miRNAs in the serum, plasma or archived material are valuable as diagnostic biomarker [9, 10].

MiRNA214 (miR-214) lies within the *DNM3*, which is described in the human q24.3 arm, it is approximately 6 kb apart [11, 12]. Accumulation of evidence have demonstrated that abnormal regulation of miR-214 can be causative for a variety of human tumors, including hepatoblastoma, hepatocellular, gastric, esophageal squamous cell carcinoma (ESCC) lung, breast, osteosarcoma, pancreatic cervical, prostate, ovarian, bladder and melanoma cancer [8]. Therefore, miR-214 has reciprocal actions in various tumor tissues that provide insight into its complex function in multiple cancer tissues with regard to both tumor suppression and tumorigenesis.

In this study, we mainly focus on the potential clinical significance of miR-214 as prognostic biomarkers for different types of cancer. We performed the systematic evaluation of the data available from studies published in this field with the main aim of assessment the role of miR-214 as a prognostic biomarker in various carcinomas.

## RESULTS

### Summary of the included studies

**Figure 1:**
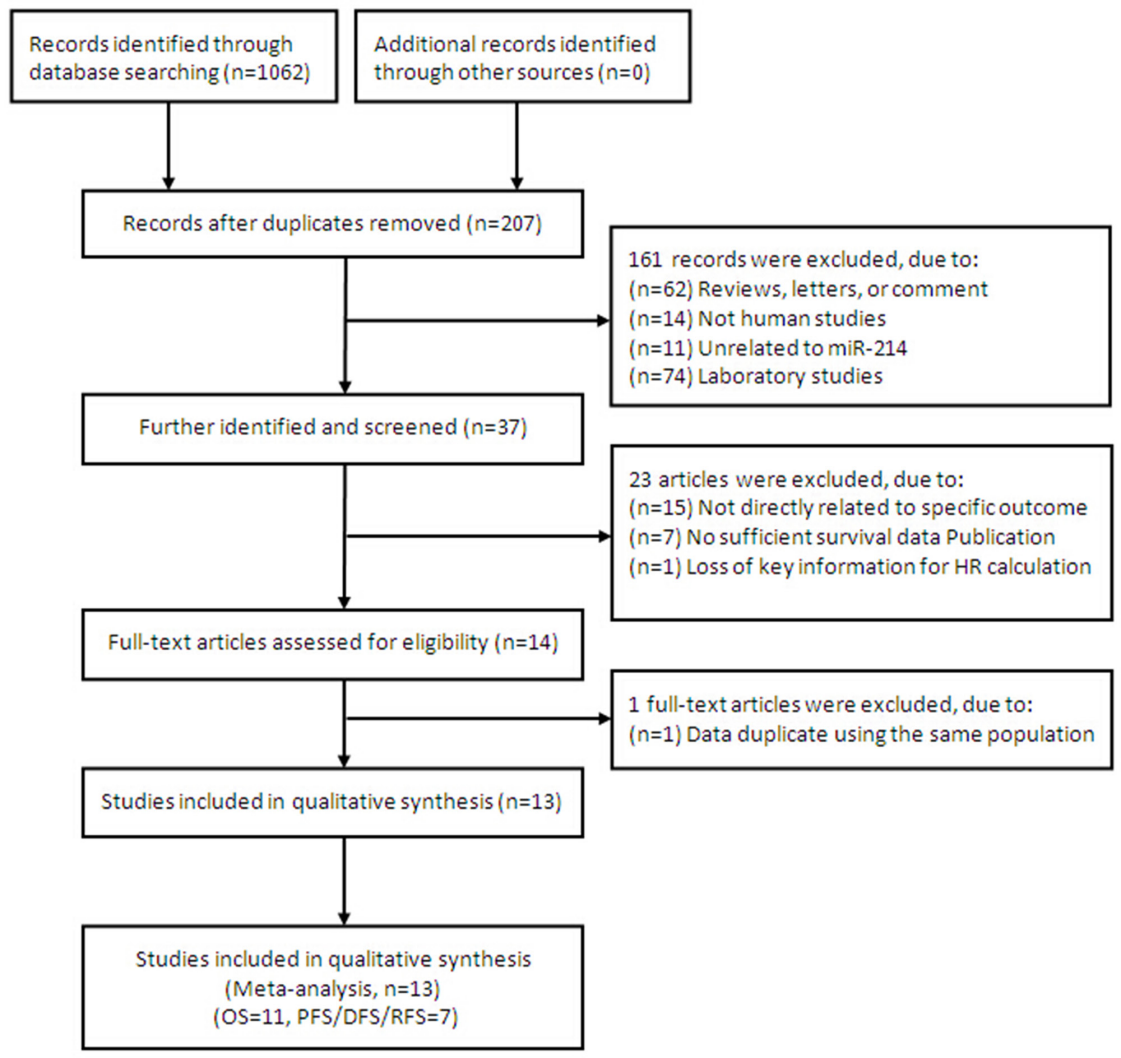
Flow chart summarizing the selection of eligible studies

After the primary literature search in database, a total of 1,062 records for miR-214 were retrieved and a flow diagram is shown in Figure 1. After duplicated studies were excluded, 207 studies were remained. According to the inclusion and exclusion criteria, 161 studies were further removed based on title and abstract screening. Fourteen potentially relevant studies were identified for full-text review as eligible, and one article [13] was further excluded because the sample is urine from bladder cancer patients, and data duplicate using the same population [25]. A final total of 13 studies [4, 14–25], 11 for OS [4, 14–20, 22, 24, 25], 7 for PFS/DFS/RFS [4, 14, 16, 20, 21, 23, 24], respectively, were considered in evidence synthesis.

The baseline characteristics of the eligible studies are summarized in Table 1. The included studies were published from 2010 to 2016 and included a total of 1256 patientswith OS data and 622 patients with DFS/PFS/RFS data from China, America, British, Latvia, Singapore, Italy and Japan. The patients according to their ethnic background were classified Asian or Caucasian. The types of carcinomas included esophageal squamous cell carcinoma (ESCC), bladder cancer, gastric cancer hepatocellular carcinoma (HCC), myeloma, pancreatic cancer, lymphoma, breast cancer, colorectal cancer, gliomas, osteosarcoma, ovarian cancer.

**Table 1:** Clinicopathological characteristics of eligible studies

Author	Year	Country	Ethnicity	Number		Histology	TNM stage	Sample	Assay	Follow-up (months)	Cut-off	Survival analysis	Hazard ratios
				OS	DFS/PFS/RFS								
Hao [14]	2016	China	Asian	108	108	Myeloma	I-III	Serum	qRT-PCR	100	Normal	OS/PFS	SC
Ali [15]	2016	America	Caucasian	35		Pancreatic Cancer	I-IIB	Frozen tissue	qRT-PCR	70	Normal	OS	SC
Wang [4]	2015	China	Asian	106	106	Bladder Cancer	I-IV	Frozen tissue	qRT-PCR	80	Median	OS/RFS	HR/SC
Lim [16]	2015	British	Caucasian	112	112	Lymphoma	I-IV	Frozen tissue	qRT-PCR	126	Normal	OS/PFS	SC
Kalniete [17]	2015	Latvia	Caucasian	50		Breast cancer	I-IV	Frozen tissue	qRT-PCR	84	Median	OS	SC
Chen [18]	2014	China	Asian	99		Colorectal Cancer	I-IV	Frozen tissue	Microarray	84	Normal	OS	HR/SC
Wang [19]	2014	China	Asian	108		Gliomas	I-IV	Frozen tissue	qRT-PCR	60	Median	OS	HR/SC
Wang (a) [20]	2013	China	Asian	92	92	Osteosarcoma	I- II	Frozen tissue	qRT-PCR	133	Median	OS/PFS	HR/SC
Wang (b) [21]	2013	China	Asian		65	HCC	I-III	Frozen tissue	qRT-PCR	56	Median	DFS	HR/SC
Zhou [22]	2013	China	Asian	104		ESCC	NG	Frozen tissue	qRT-PCR	36	Mean	OS	SC
Xia [23]	2012	Singapore	Asian		50	HCC	NG	Frozen tissue	qRT-PCR	120	Median	DFS	SC
Marchini [24]	2011	Italy	Caucasian	89	89	Ovarian cancer	I-IV	Frozen tissue	qRT-PCR	156	Normal	OS/PFS	HR
Ueda [25]	2010	Japan	Asian	353		Gastric cancer	I-IV	Frozen tissue	qRT-PCR	84	Median	OS	HR/SC

TNM, tumor node metastasis; HCC, hepatocellular carcinoma; ESCC, esophageal squamous cell carcinoma; qRT-PCR, quantitative real-time PCR; OS, overall survival; PFS, progressive free survival; DFS, disease free survival; RFS, recurrence free survival; SC, survival curve.

Tissue specimens were used in 12 studies and serum was used in one study. Quantitative real-time polymerase chain reaction (qRT-PCR) was conducted in 12 studies and Microarray was used in the remaining one study. Notably, the cutoff values of miR-214 were different in the studies, most with median or mean. Details of the characteristics in the final synthesis were summarized in Table 1.

### Qualitative assessment

According to the QUIPS for estimation of quality in prognostic studies, the evaluation results of each item with potential bias were presented as “yes”,“partly”, “no” or “unsure” in Table 2. The key characteristics of baseline were adequately presented and the adopted statistical analyses were appropriate in all eligible studies. Among 13 studies, eight studies were prospective cohort researches (level of evidence: 1b) whereas 5 were retrospective designs (level of evidence: 2b) (Table 2). The methodological quality scores of included studies based on the NOS ranged from 5 to 8, the average scores of studies were 6.92 (Table 2, Supplementary Table 1).

**Table 2:** Quality assessment of included studies based on the Quality In Prognosis Studies (QUIPS)

Study	Quality evaluation of prognosis study						Total score^a^	Level of evidence^b^
	Study participation	Study attrition	Prognostic factor measurement	Outcome measurement	Study confounding	Statistical analysis and reporting		
Hao 2016 [14]	Yes	Yes	Yes	Partly	Partly	Yes	**6**	**2b**
Ali 2016 [15]	Yes	Yes	Partly	Partly	Partly	Yes	**6**	**2b**
Wang 2015 [4]	Yes	Yes	Yes	Yes	Partly	Yes	**7**	**1b**
Lim 2015 [16]	Yes	Yes	Yes	Partly	Partly	Yes	**8**	**1b**
Kalniete 2015 [17]	Yes	Yes	Yes	Partly	Partly	Yes	**7**	**1b**
Chen 2014 [18]	Yes	Yes	Yes	Yes	Partly	Yes	**7**	**1b**
Wang 2014 [19]	Yes	Yes	Yes	Yes	Partly	Yes	**7**	**1b**
Wang (a)2013 [20]	Yes	Yes	Yes	Yes	Partly	Yes	**8**	**1b**
Wang (b) 2013 [21]	Yes	Yes	Yes	Partly	Partly	Yes	**7**	**2b**
Zhou 2013 [22]	Yes	Yes	Partly	Partly	Partly	Yes	**5**	**2b**
Xia 2012 [23]	Yes	Yes	Partly	Partly	Partly	Yes	**6**	**2b**
Marchini 2011 [24]	Yes	Yes	Yes	Yes	Partly	Yes	8	**1b**
Ueda 2010 [25]	Yes	Yes	Yes	Yes	Partly	Yes	8	**1b**

^**a**^ Quality assessment of included studies based on the Newcastle–Ottawa Scale.

^**b**^ The levels of evidence were estimated for all included studies with the Oxford Centre for Evidence Based Medicine criteria.

### Evidence synthesis and test of heterogeneity

**Figure 2:**
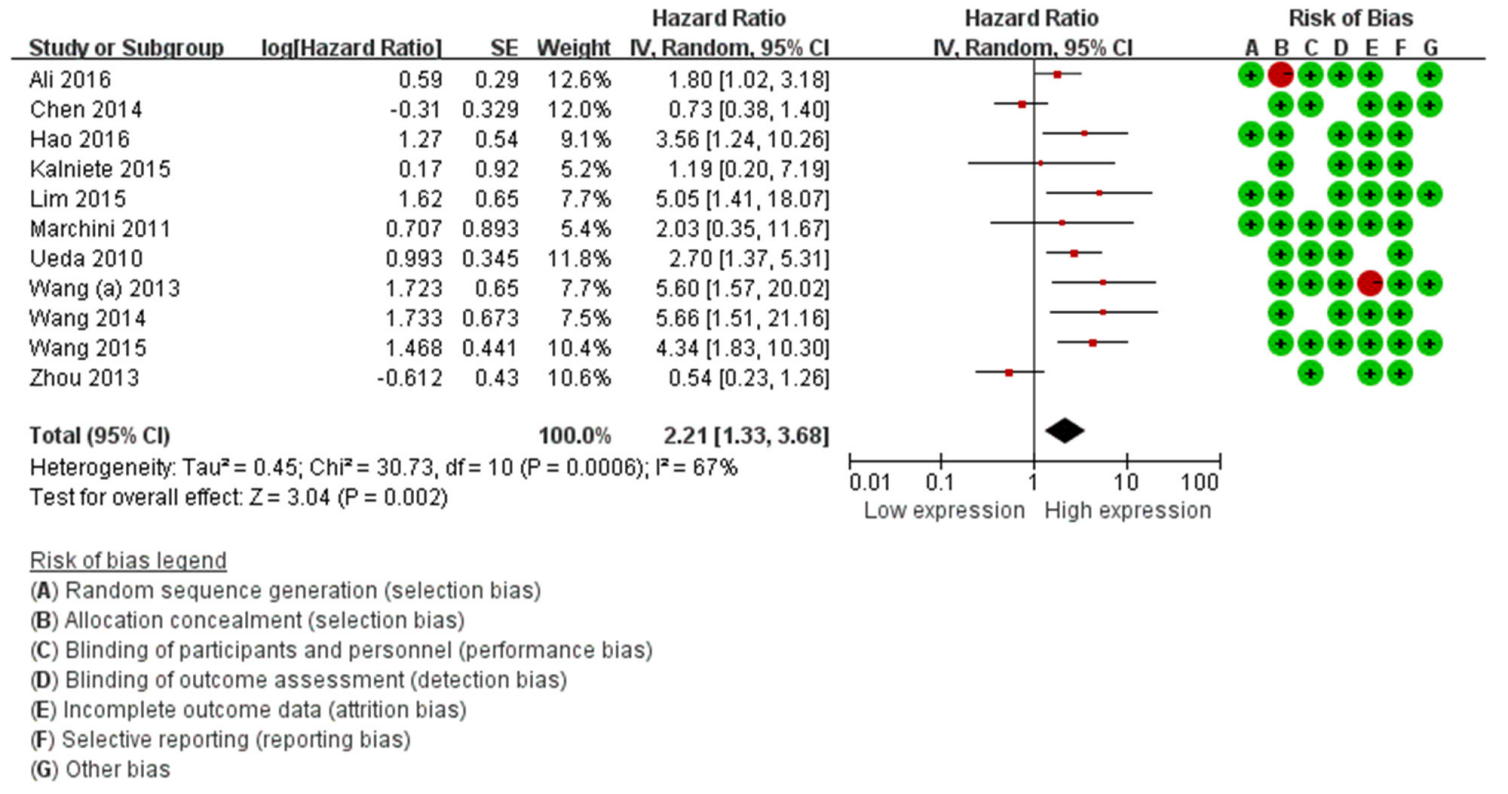
Forest plots of the relationship between elevated miR-214 level and OS The squares and horizontal lines correspond to the study-specific OR and 95% CI. The area of the squares reflects the study specific weight. The diamond represents the pooled OR and 95% CI.

The main results of the pooled analyses and the heterogeneity tests are shown in Table 3. Eleven articles evaluated OS for miR-214, a statistically significant risk association was observed in the overall pooled analysis (HR=2.21, 95%CI: 1.33-3.68, *P*=0.00), (Figure 2). In the subgroup analysis by ethnicity, there was a significant association in Asian (HR=2.27, 95%CI: 1.09-4.73, *P*=0.00) and Caucasian patients (HR=2.04, 95%CI: 1.47-3.30, *P*=0.00). Further, subgroup analysis stratified by cancer type suggested a significant positive relationship between miR-214 expression and OS was revealed in other cancers (excluding digestive tract cancer, DTC) (HR=2.90, 95%CI: 2.02-4.15, *P*=0.00).

**Table 3:** Main results of pooled HRs in the meta-analysis

Comparisons	Heterogeneity test			Summary HR (95% CI)	Hypothesis test		Studies
	*Q*	*P*	*I*^*2*^(%)		*Z*	*P*	
*Total*							
OS	30.73	<0.01	67	2.21(1.33,3.68)	3.04	<0.01	11
DFS/PRS/RFS	33.24	<0.01	82	1.73(0.78,3.83)	1.36	0.18	7
*Ethnicity*							
OS							
Asian	28.12	<0.01	69	2.27(1.09,4.73)	2.19	0.03	7
Caucasian	2.48	0.48	0	2.04(1.47,3.30)	2.92	<0.01	4
DFS/PRS/RFS							
Asian	29.07	<0.01	68	1.52(0.57,4.05)	0.83	0.41	5
Caucasian	1.06	0.30	6	2.74(1.20,6.25)	2.39	0.02	2
*Cancer subtypes*							
OS							
DTC	10.99	<0.01	68	1.10(0.73,1.65)	0.45	0.65	3
Other cancers	7.51	0.38	7	2.90(2.02,4.15)	5.81	<0.01	8
DFS/PRS/RFS							
HCC	1.98	0.16	49	0.39(0.22,0.69)	3.26	<0.01	2
Other cancers	4.11	0.39	3	3.11(2.00,4.84)	5.04	<0.01	5

DTC, digestive tract cancer, including colorectal cancer, oral cavity, esophageal squamous cell carcinoma and hepatocellular carcinoma (ESCC).

**Figure 3:**
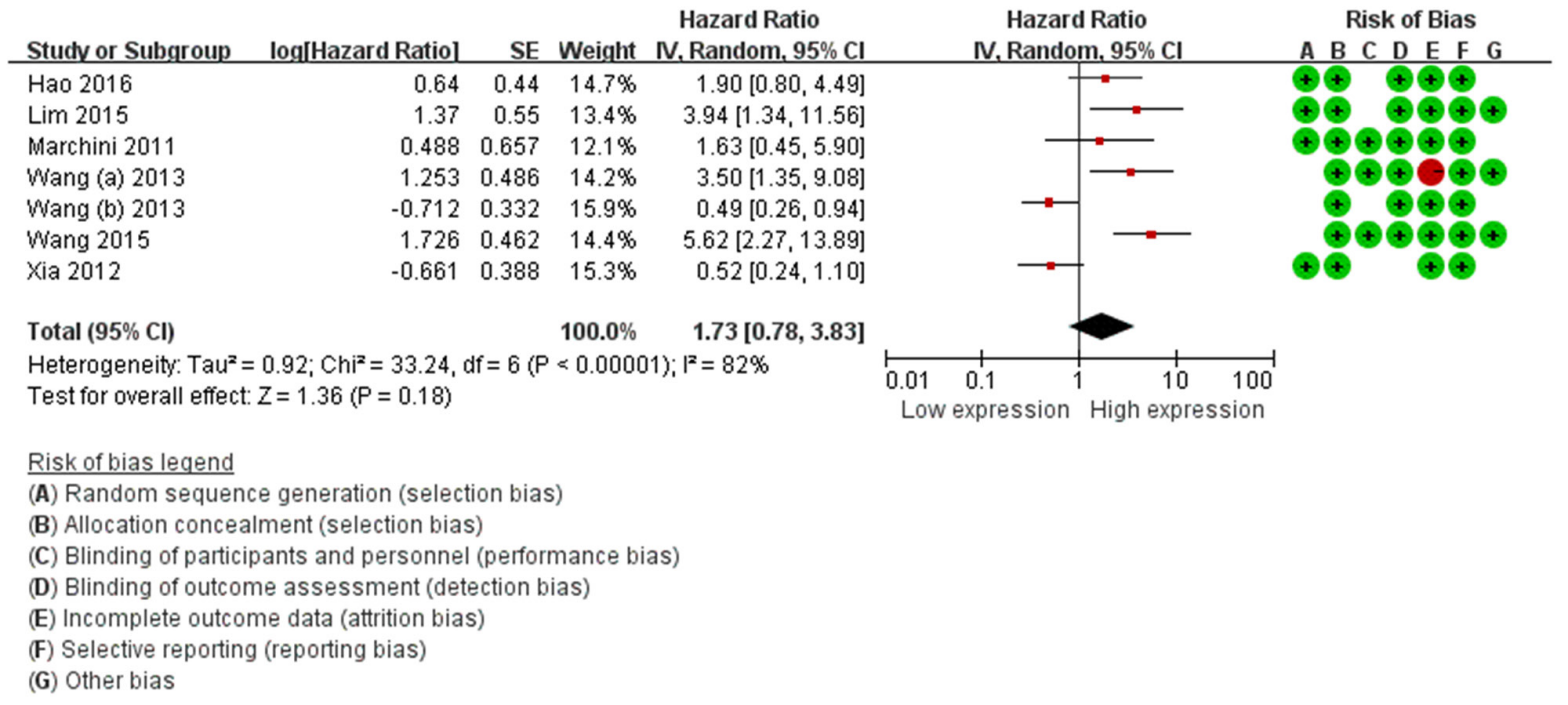
Forest plots of the relationship between elevated miR-214 level DFS/PFS/RFS

For the DFS/PFS/RFS, we failed to find associations between miR-214 expression and predicted survival (HR=1.73, 95%CI: 0.78-3.83, *P*=0.18) (Table 3, Figure 3). We performed subgroup analysis according to ethnicity, a significant relationship between miR-214 expression and DFS/PFS/RFS was observed in Caucasian patients (HR=2.74, 95%CI: 1.20-6.25, *P*=0.02). In addition, subgroup analysis was further carried out according to cancer type, the result showed that a higher expression level of miR-214 significantly predicted favorable DFS/PFS/RFS in hepatocellular carcinoma (HCC) (HR=0.50, 95%CI: 0.31-0.82, *P*=0.00) (Table 3).

### Heterogeneity analysis

To explain the heterogeneity, we assessed the source of heterogeneity. Meta regression in Stata 13.3MP was used to evaluate by publication year, cancer type, ethnic, language, assay, sample size (100 as the boundary), quality (Based on NOS, ≥7 or <7). It was detected that the main results were not affected by above characteristics (Table 4).

**Table 4:** The results of heterogeneity test

Comparisons	Coef.	Std. Err.	*t*	*P*	95% CI
Publication year	-0.209	0.919	-0.02	0.983	-2.271-2.229
Cancer type	-0.426	0.140	-0.31	0.770	-0.384-0.299
Ethnic	0.402	1.297	0.31	0.767	-2.772-3.576
Language*	-	-	-	-	-
Assay	1.403	1.446	0.97	0.369	-2.135-4.941
Sample size	0.441	1.322	0.33	0.750	-2.984-1.196
Quality	-0.849	0.854	-1.05	0.336	-2.984-1.196

*Language dropped because of collinearity.

### Sensitivity analysis and publication bias

**Figure 4:**
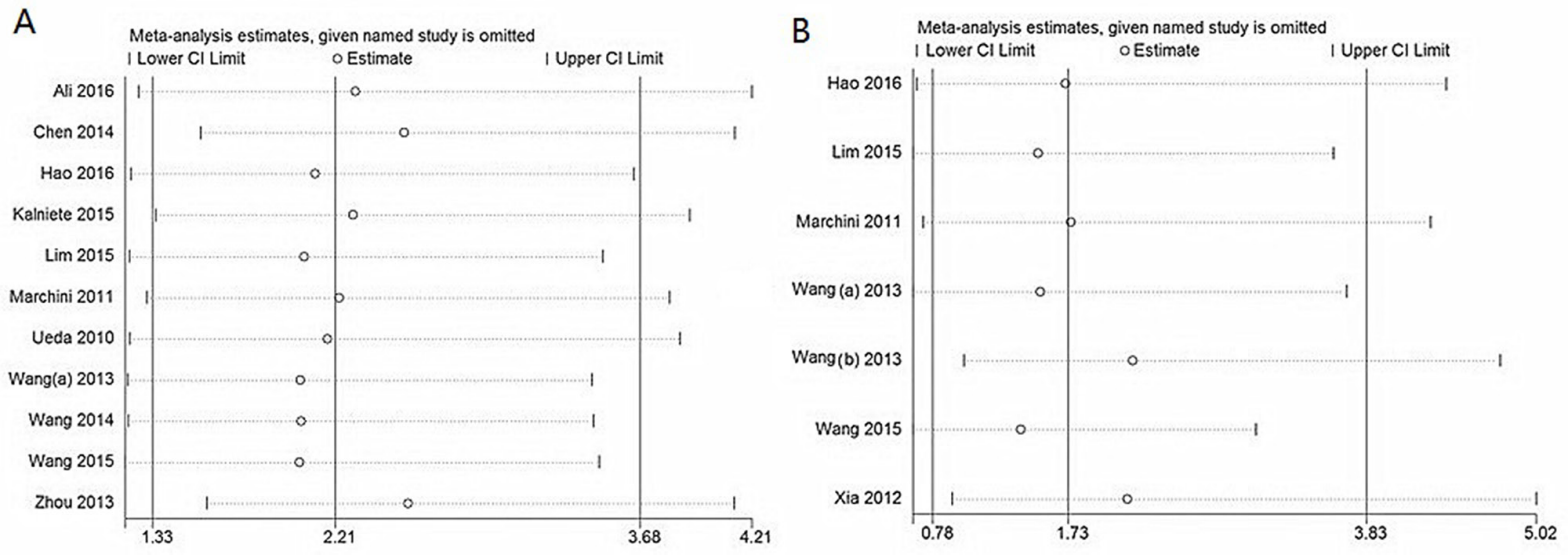
Sensitivity analysis for OS **(A)** and DFS/PFS/RFS **(B).**

**Figure 5:**
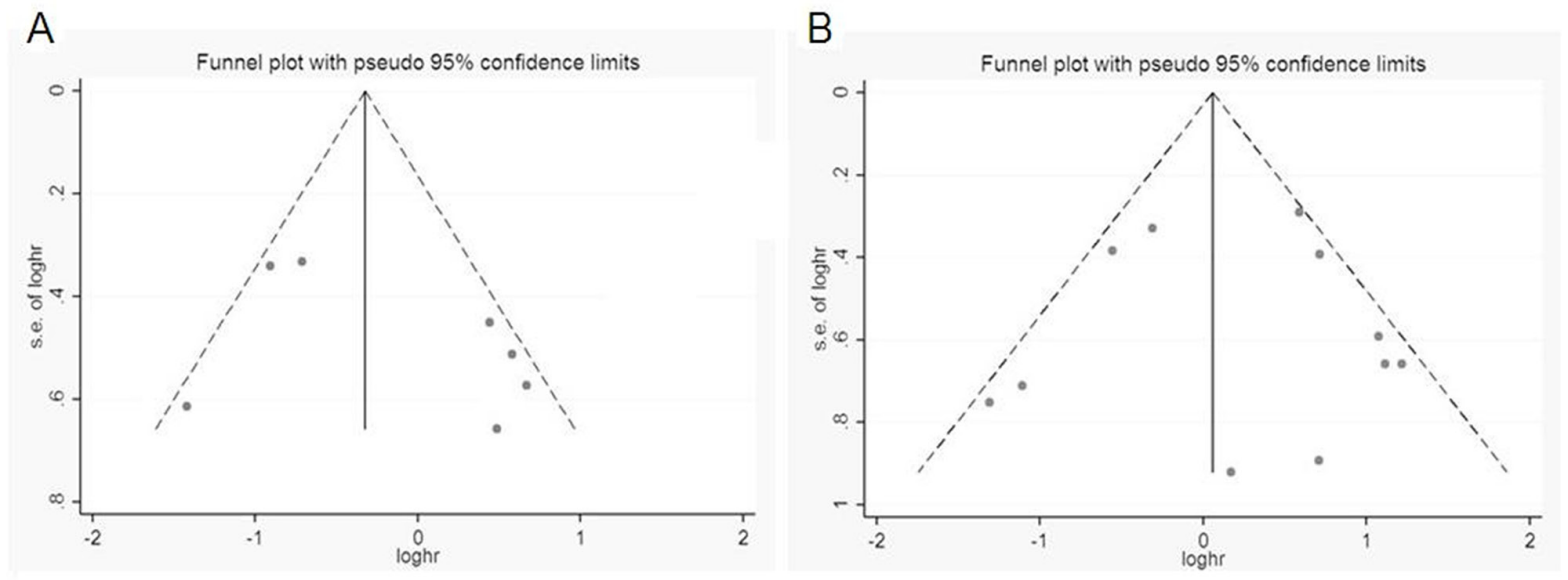
Funnel plot for publication bias analysis **(A)** OS and **(B)** DFS/PFS/RFS. The vertical line in the funnel plot indicates the fixed-effects summary estimate, whereas the sloping lines indicate the expected 95%CI for a given SE.

Sensitivity analysis data sets showed that the pooled HRs were not significantly influenced by omitting individual study (Figure 4). Begg’s funnel plot and Egger’s test were conducted to detect of publication bias (Table 5). The shape of the funnel plot did not indicate any visual evidence of the asymmetry (Figure 5), indicating that the results were statistically robust in this study.

**Table 5:** Publication bias of miR-218 for Begg’s test and Egger’s test

Comparisons	Begg’s test		Egger’s test		
	*z*	*p*	*t*	*p*	95% CI
OS	0.08	0.938	0.43	0.676	-3.730-5.492
DFS/PRS/RFS	0.60	0.548	1.61	0.169	-3.164-13.752

## DISCUSSION

Evidence has been increasing that many target genes of miR-214 which regulate several biological processes, such as tumorigenesis, differentiation and angiogenesis [8, 26, 27]. Hence, biomarkers of tumor are important and necessary tools in detection and clinical practice. The validated association with biological processes or outcomes, and combine good measurability are essential for useful biomarkers, when applied to clinical practice, it should support clinical decision making [28]. Despite innovative discoveries and intensive technological analysis in the development of the early stage biomarkers and translational research, miR-214 has not been validated in the clinical setting.

Substantial data illustrates the dysregulation of miR-214 in different types of cancer [29], it indicates that different patterns of miR-214 expression may play a role in the carcinogenesis [30]. Identification of dysregulated miRNAs in different stages of cancers or in various tumors types could provide novel insight into the potency of miR-214 as a diagnostic and prognostic biomarker in different cancers [31].

In response to the need for independently prognostic molecular markers for cancers, we conducted this pooled analysis of published literature to identify the miR-214 for which the data support validation as prognostic biomarkers of various cancer outcomes.

To our knowledge, this report is the first to critically examine available literature and identify the prognostic role of miR-214 in different types of cancer. Therefore, we gathered the available evidence from all relevant studies to evaluate the prognostic values of miR-214. The results demonstrated that expression of miR-214 was significantly correlated with OS in cancers (HR=2.21, 95%CI: 1.33-3.68, *P*=0.00). Our stratified analysis suggested a closer relationship between rising miR-214 levels and poor survival in Asians (HR=2.27, 95%CI: 1.09-4.73, *P*=0.00) and Caucasians (HR=2.04, 95%CI: 1.47-3.30, *P*=0.00). Due to the included studies used a variety of indices to evaluate tumor progression, such as DFS, PFS or RFS, we combined these indices to evaluate the prognostic value of miR-214. For studies evaluating DFS/PFS/RFS, no correlation of miR-214 expression with DFS/PFS/RFS in cancers (HR=1.73, 95%CI: 0.78-3.83, *P*=0.18). However, in our subgroup analysis, we found that high miR-214 expression significantly predicted favorable DFS/PFS/RFS (HR=2.57, 95%CI: 1.37-4.81, *P*=0.00) in HCC group, it may be a potential prognostic biomarker in HCC.

MiRNAs display different levels of expression and predictive values across various ethnic groups [32]. Several studies have identfed reduced miRNA-214 expression in HCC [33], and unusual hypervascularity is a hallmark of HCC [34]. Downregulaton of miRNA-214 induces hepatoma-derived growth factor expression and secreton, thereby stmulatng vascular endothelial cells for angiogenesis [35]. Further, downregulaton of miRNA-214 induces the expression of enhancer of β-catenin (directly or indirectly through EZH2) and zeste homolog 2 (EZH2) (directly) [36]. The human β-catenin signaling pathway activate plays an activation function in the proliferaton and invasion of HCC cells [23]. In this sense, miR-214 is a promising biomarker for early detection and cancer prognosis in Caucasians. The role of miR-214 in HCC prognosis remains unclear, although the included studies suggested that miR-214 could be a suitable prognostic biomarker for HCC. Therefore we strongly suggest conducting more prognostic studies for abnormal expression of miR-214 in HCC. These findings have raised a question about whether miR-214 paly dual function role as both a tumor promoter and suppressor, it is partly dependent on the specific signaling pathways in each of the different types of cancer [37].

Irrespective of the mechanism or clinical verification of miR-214, the results suggest that miR-214 can be used as a predictive biomarker of cancer prognosis in Caucasians. However, we make this conclusion cautiously, and some details must be addressed for practical value of mir-214 prognosis.

Firstly, the reliability of our results is questionable in light of the number of eligible studies for OS and DFS/RFS/RFS. Secondly, the number of certain tumor type of included prognostic studies was not sufficient, which might impact the statistical power of analysis. Therefore, further well-designed clinical studies with larger sample sizes in different ethnic groups should be conducted. Thirdly, due to not all the survival data of the eligible studies were given directly, some data was extracted from survival curves. These calculated HRs with corresponding 95%CIs might be brought several tiny errors. Fourthly, most included studies use median or mean value as the cut-off value, but the actual value was different, the lack of a golden standard, a clear definition should be made about the cutoff value of miR-214 level for survival risk. Finally, although we find no evidence of publication bias in the present study, cautions should be taken, because the journals tend to publish positive results could also make publication bias, and all included studies were published in English, which could definitely cause language bias.

In conclusion, our data demonstrated that high miR-214 could be a promising biomarker for prognosis prediction of cancer. However, the current data are insufficient, the clinical significance of the expression in malignant tumor still need to be determined in the future.

## MATERIALS AND METHODS

Ethics committee is not applicable in the present study.

This study was performed according to the guidelines of the Meta-analysis of Observational Studies in Epidemiology group (MOOSE) issued by Stroup et al [38]. and Preferred Reporting Items for Systematic Review and Meta-analyses (PRISMA) criteria [39].

### Literature search strategy

Literature searches of PubMed, Embase, Web of Science databases, Chinese National Knowledge Infrastructure (CNKI) and Wanfang database were carried out from January 1st, 1989 through Sep 26th, 2016 with the following terms: ‘microRNA-214’ or ‘miR-214’ and ‘neoplasms’ or ‘cancer’. Electronic search restrictions were set for the English and Chinese language. In addition, reference lists of retrieved publications were examined manually to further identify missing relevant articles.

### Inclusion and exclusion criteria

The inclusion criteria for the studies were as follows: (i) the full-text article was available in English or Chinese; (ii) the subjects were patients with any type of cancer; (iii) miR-214 expression was measured in tumor tissue or serum and (iv) reporting the survival outcome or the correlation between miR-214 expression and the clinical variables.

The exclusion criteria included: (i) reviews, letters or laboratory studies; (ii) non English or Chinese articles; (iii) overlapping database or duplicated studies using the same population and (iv) lacked key information regarding survival outcomes, such as hazard ratios (HRs) or 95% confidence intervals (95%CIs) or unable to calculate such parameters.

The retrieved articles were assessed for inclusion by FJD and YJF independently and all disagreements were resolved via discussion.

### Data extraction

Two investigators (FJD, WGL) evaluated and extracted the data independently from all eligible studies under the guideline of a critical review checklist. Data for analyses, including first author, publication year, origin country, histological classification, TNM stage, sample type and size, detection method, follow-up and cutoff value, HRs of miR-214 for overall survival (OS) and/or progressive free survival (PFS), disease free survival (DFS), recurrence free survival (RFS) and the corresponding 95% CIs.

If not available, data were calculated following Tierney et al.’s method [40]. If discrepancies existed, disagreements were resolved via discussion.

### Evaluation of study quality

The methodological quality of each study was systematically assessed according to a critical review checklist of the Dutch Cochrane Centre proposed by MOOSE to ensure their quality [38].

The levels of evidence were estimated for all included studies with the Oxford Centre for Evidence Based Medicine criteria [41]. Quality assessment criteria were utilized to evaluate methodological quality of included studies based on Newcastle-Ottawa Scale (NOS) for quality of case-control and cohort studies [42]. In addition, the specific Quality In Prognosis Studies (QUIPS) was estimated according to the approach of Hayden et al [43]. The evaluated items with potential bias included study participation, study attrition, prognostic factor measurement, outcome measurement, study confounding, and statistical analysis and reporting. The assessments were processed independently by two reviewers and the final decision was achieved by consensus.

### Statistical analysis

Statistical analysis was performed using RevMan software version 5.3.5 (Cochrane Collaboration, Oxford, UK) and STATA software version 13.1MP (StataCorp, College Station, TX, USA).

All of the HRs and corresponding 95%CIs were used to calculate the pooled HR. Cochran’s *Q* test and Higgin’s *I*^2^ statistic was used to measure between-study heterogeneity. If heterogeneity did exist (*P*_heterogeneity_<0.05 or *I*^2^>50%), random-effects model (DerSimonian and Laird method) [44] was applied to calculate pooled HR, and meta-regression were further applied to investigate sources of heterogeneity [45]. If not, fixed-effects model (Mantel-Haenszel method) [46] was used. The stratified analysis was conducted by ethnicity (Asians, Caucasians) and cancer type.

One-way sensitivity analyses were performed, and then by omitting each study at a time to assess the quality and consistency of the pooled results.

Publication bias was evaluated using Begg’s test (rank correlation test) [47] and Egger’s test (weighted linear regression test) [48]. If a publication bias did exist, the trim and fill method [49] was used to adjust the results

The significance of merged HR was dependent on the Z-test, *P* values less than 0.05 (*P*<0.05) was considered statistically significant, all *P* values were two-sided.

## SUPPLEMENTARY MATERIALS TABLE



## Abbreviations

miR-214: microRNA-214
miRNAs: microRNAs
HRs: hazard ratios
Cis: confidence intervals
DFS: disease free survival
PFS: progressive free survival
RFS: recurrence free survival
