# Supplementary material for: Prognostic value of the microRNA-214 in multiple human cancers: a meta-analysis of observational studies
Source: Oncotarget. 2017 May 6;8(43):75350–60. doi: 10.18632/oncotarget.17642 (PMC5650425; doi:10.18632/oncotarget.17642)
Supplement: Supplementary file 1 [file oncotarget-08-75350-s001.pdf]

Prognostic value of the microRNA-214 in multiple human cancers: a meta-analysis of observational studies

SUPPLEMENTARY TABLE

Supplementary Table 1: Quality assessment of included studies based on the Newcastle–Ottawa Scale for assessing the quality of cohort studies.

Study	Selection (score)				Comparability (score)	Exposure (score)			Total Score[b]
	Represen-tativenes Of the exposed cohort	Selection of the non-exposed cohort	Ascertain-ment of exposure	Outcome of interest was not present at start of study	Based on the design or analysis[a]	Assessment of outcome	Follow-up long enough for outcomes to occur	Adequacy of follow-up of cohorts	
Hao 2016 [14]	1	0	1	1	1	0	1	1	**6**
Ali 2016 [15]	1	1	1	1	0	0	1	1	**6**
Wang 2015 [4]	1	1	1	1	1	1	1	1	**7**
Lim 2015 [16]	1	1	1	1	2	0	1	1	**8**
Kalniete 2015 [17]	1	0	0	1	2	1	1	1	**7**
Chen 2014 [18]	1	0	0	1	2	0	1	1	**7**
Wang 2014 [19]	1	0	1	1	2	0	1	1	**7**
Wang (a)2013 [20]	1	0	1	1	2	1	1	1	**8**
Wang (b) 2013 [21]	1	0	1	1	2	0	1	1	**7**
Zhou 2013 [22]	1	0	1	1	0	0	1	1	**5**
Xia 2012 [23]	1	0	1	1	1	0	1	1	**6**
Marchini 2011 [24]	1	0	1	1	2	1	1	1	**8**
Ueda 2010 [25]	1	0	1	1	2	1	1	1	**8**

[a] When there was no statistical significance in the response rate between case and control groups by using a chi-squared test ($P > 0.05$), one point was awarded.
[b]Total score was calculated by adding up the points awarded in each item.
